# Effectiveness of vortioxetine in real-world clinical practice: French cohort results from the global RELIEVE study

**DOI:** 10.1192/j.eurpsy.2022.1454

**Published:** 2022-09-01

**Authors:** M. Polosan, M. Rabbani, K. Simonsen, H. Ren

**Affiliations:** 1 GIN, Inserm U836, Grenoble, France; 2 Lundbeck France, Medical Affairs, Paris, France; 3 H.Lundbeck A/S, Medical Affairs, Valby, Denmark

**Keywords:** real world evidence, vortioxetine, Depression, effectiveness

## Abstract

**Introduction:**

Major depressive disorder (MDD) affects around 10% of the French population annually and significantly impacts patient functioning. Efficacy of vortioxetine was demonstrated in randomised controlled trials, data on its real-world performance is needed.

**Objectives:**

To describe the effectiveness and safety of vortioxetine in real-world setting from patients enrolled from France in the global RELIEVE study.

**Methods:**

RELIEVE was a prospective, multi-national, observational study of outpatients initiating vortioxetine treatment for MDD at physician’s discretion. Data were collected at routine clinical visits. Here we present the outcomes of treatment of patients in France. The primary outcome was functioning measured by SDS. Secondary outcomes included depressive symptoms measured by PHQ-9, cognitive symptoms measured by PDQ-5 and DSST. Changes from baseline to month 6 were estimated with a linear mixed model of repeated measures approach.

**Results:**

A total of 184 patients (mean age, 50.2 years, 65% female, 67.9% of patients had at least one comorbidity) were enrolled from France and included in the analysis. Mean(SD) SDS total score, PHQ-9, PDQ-5 scores at baseline were 21.1(5.4), 17.5(4.7) and 11.7(4.4), the scores(SE) decreased by 10.9(0.59), 9.3(0.48) and 6.1(0.37) from baseline to month 6. Mean(SD) DSST improved from 41.6(15.2) at baseline to 49.1(19.0) at month 6. Safety and tolerability profile of vortioxetine was in line with previous studies.

**Conclusions:**

Sustained improvements in overall functioning, depressive symptoms, cognitive function were observed in patients treated with vortioxetine in a real-world setting, which provided further evidence of effectiveness and safety of vortioxetine in a broad MDD population in France.

**Disclosure:**

M. Rabbani is an employee of Lundbeck France. K. Simonsen and H. Ren are employees of H. Lundbeck A/S.

